# A Finite Element Mesh Regrouping Strategy-Based Hybrid Light Transport Model for Enhancing the Efficiency and Accuracy of XLCT

**DOI:** 10.3389/fonc.2021.751139

**Published:** 2022-01-17

**Authors:** Yanqiu Liu, Xiangong Hu, Mengxiang Chu, Hongbo Guo, Jingjing Yu, Xiaowei He

**Affiliations:** ^1^ Key Laboratory for Radiomics and Intelligent Sense of Xi’an, Northwest University, Xi’an, China; ^2^ School of Information Sciences and Technology, Northwest University, Xi’an, China; ^3^ Network and Data Center, Northwest University, Xi’an, China; ^4^ School of Physics and Information Technology, Shaanxi Normal University, Xi’an, China

**Keywords:** X-ray luminescence computed tomography, mesh regrouping, hybrid light transport model, Eu^3+^-based nanophosphors, inverse reconstruction

## Abstract

X-ray luminescence computed tomography (XLCT) is an emerging hybrid imaging modality in optical molecular imaging, which has attracted more attention and has been widely studied. In XLCT, the accuracy and operational efficiency of an optical transmission model play a decisive role in the rapid and accurate reconstruction of light sources. For simulation of optical transmission characteristics in XLCT, considering the limitations of the diffusion equation (DE) and the time and memory costs of simplified spherical harmonic approximation equation (*SP_N_*), a hybrid light transport model needs to be built. DE and *SP_N_* models are first-order and higher-order approximations of RTE, respectively. Due to the discontinuity of the regions using the DE and *SP_N_* models and the inconsistencies of the system matrix dimensions constructed by the two models in the solving process, the system matrix construction of a hybrid light transmission model is a problem to be solved. We provided a new finite element mesh regrouping strategy-based hybrid light transport model for XLCT. Firstly, based on the finite element mesh regrouping strategy, two separate meshes can be obtained. Thus, for DE and SP*_N_* models, the system matrixes and source weight matrixes can be calculated separately in two respective mesh systems. Meanwhile, some parallel computation strategy can be combined with finite element mesh regrouping strategy to further save the system matrix calculation time. Then, the two system matrixes with different dimensions were coupled though repeated nodes were processed according to the hybrid boundary conditions, the two meshes were combined into a regrouping mesh, and the hybrid optical transmission model was established. In addition, the proposed method can reduce the computational memory consumption than the previously proposed hybrid light transport model achieving good balance between computational accuracy and efficiency. The forward numerical simulation results showed that the proposed method had better transmission accuracy and achieved a balance between efficiency and accuracy. The reverse simulation results showed that the proposed method had superior location accuracy, morphological recovery capability, and image contrast capability in source reconstruction. *In*-*vivo* experiments verified the practicability and effectiveness of the proposed method.

## 1 Introduction

X-ray luminescence computed tomography (XLCT) is an emerging hybrid imaging modality in optical molecular imaging (1–4). Compared with other optical molecular imaging modalities, e.g., fluorescence molecular tomography (FMT) (5) and bioluminescence tomography (BLT) (6), XLCT combines optical imaging with CT imaging and realizes molecular imaging and functional imaging simultaneously. In the imaging system, high-energy X-ray excites nanophosphors *in vivo*, and optical photons are emitted and captured by a highly sensitive charge-coupled device (CCD) camera (7–9). Based on the properties of X-rays, XLCT has the advantages of weak autofluorescence and high spatial resolution, which has attracted more attention and has been widely studied (10–14).

In related studies on XLCT at present, nanophosphors inside an imaging object, irradiated by X-rays, emit visible or near-infrared (NIR) light that can be detected by an optical detector (15). According to literature research results, Eu^3+^-based [Eu_2_O_3_ (13), Y_2_O_3_:Eu^3+^ (16), GOS:Eu^3+^ (17)] and Tb^3+^-based [Gd_2_O_2_S:Tb^3+^ (18)] nanometer materials are often used as X-ray excitable nanophosphors. Yang et al. studied that Eu^3+^ has several weak emission peaks at 533, 580, 586, 592, 599, 650, and 706 nm, and 610 nm is the highest emission peak under ultraviolet excitation (259 nm), which shows a strong red emission (19). Chen et al. verified that the emission peaks in the emission luminescence spectrum of Gd_2_O_2_S:Tb^3+^ locate at wavelengths of 545, 585, and 620 nm, and the highest peaks locate at wavelengths of 545 nm (20). It is consistent with the conclusion on the luminescence properties of Tb^3+^ doping and it corresponds to green emission (21). Due to reduced tissue-scattering effect resulting from longer wavelength, Eu^3+^-based nanometer material is more commonly used as X-ray excitable nanophosphors. Based on this, the study of the light transport model should focus on the luminescence characteristics of Eu^3+^. In XLCT, diffusion equation (DE) is most commonly used to model the photon migration in biological tissues in the studies reported so far, and in all these studies, Eu^3+^ is chosen as the luminescent particle (16, 22–24). In addition, in the research conclusions of Zhang et al., *SP*_3_ simulation is more suitable for Eu^3+^ luminescence (25).

Generally, in the study of FMT and BLT, the radiative transfer equation (RTE) has been successfully used as an accurate model for light propagation in a medium. However, in practical application, implementation of RTE is extremely complicated for complex biological tissues and consumes extensive computational time (26). Several approximation models of RTE have been studied to model the light transport in a turbid medium, such as DE, the simplified spherical harmonic approximation equation (*SP_N_*), the discrete ordinates equation (*S_N_*), the spherical harmonics equation (*P_N_*), and the phase approximation (PA) (27). However, although DE has a high computational efficiency, it only applies to biological tissues with high scattering properties (27). For Eu^3+^ luminescence performance, the red light emitted by Eu^3+^ at 610 nm passes through different organs in the non-homogeneous biological tissue, showing high scattering and non-highly scattering properties in different organs. Singly using the DE model may affect the accuracy of light propagation in non-highly scattering tissues. The higher ordered optical transmission models are shown to have improved accuracy than DE, although *SP_N_* approximation leads to a lower computational load than either *S_N_* or *P_N_* approximations, and the number of unknowns to be solved is still several times than DE (28). The higher ordered approximation is used throughout the entire domain, leading to an increase in the number of variables at each node of the FEM mesh and bringing a higher computational load. The ideal light propagation model should be established according to the performance of the actual emitted light.

In order to solve the limitations of single optical transmission models, some hybrid optical transmission models were proposed to strive for the balance between efficiency and precision. To solve the special problems of non-scattering regions, such as clear cerebrospinal fluid, stomach, and bladder, hybrid models based on radiance were proposed (29–31). The hybrid radiosity–diffusion method used the diffusion theory to analyze the scattering regions and was combined with a radiosity approach to analyze the propagation through the clear region. The hybrid *SP_N_*–radiosity method combined *SP*_3_ with the radiosity equation, which provided acceptable accuracy in the turbid medium with both low-scattering and non-scattering regions. Furthermore, to solve the problem of light transmission in non-highly scattering regions and area close to the source, several hybrid models based on DE have been studied. The hybrid Monte Carlo–diffusion method was adopted to calculate the head models, including the low-scattering region in which the light propagation obeys neither diffusion approximation nor radiosity theory. In this method, the high-scattering and low-scattering regions were modeled by diffusion approximation and the Monte Carlo method, respectively (30). The hybrid radiative transfer equation–diffusion approximation method was studied to solve the inefficiency of the DE model applied in the proximity of the collimated light sources. In detail, the light propagation in the vicinity of the laser sources was modeled by radiative-transfer equation, diffusion approximation was used elsewhere in the domain, and the accuracy of the forward model was improved compared with the conventional diffusion model (31, 32). The hybrid diffusion equation–*SP_N_* method considered the applicability of *SP_N_* and DE models in different biological tissues, and DE was employed to describe light propagation in high-scattering tissues, while *SP_N_* was used in other tissues. This method achieved a comparable accuracy and much less computation time compared with the *SP_N_* model and a much better accuracy compared with DE as well (33, 34). The studies of hybrid models offer ideas to the optical transmission model in our study.

To balance computational accuracy and efficiency of optical transmission in XLCT, we provide a new finite element mesh regrouping strategy-based hybrid light transport model (MRHM) in this paper. In this method, according to the optical properties of biological tissues, each organ of the organism is judged to apply to DE approximation or *SP*_3_ approximation based on the value of absorption and reduced scattering coefficients. According to their applicable model, organs are divided into two different regions: the nodes and tetrahedrons are rearranged according to the regions they locate at, so two independent grids are formed. DE and *SP*_3_ are used for modeling in the two grids, respectively. The two regions have corresponding correlation system matrixes, the two meshes are merged into a regrouping mesh by coupling two system matrixes, and a hybrid optical transmission model is established. In numerical simulations and mouse-based experiments, the accuracy and efficiency of our proposed method will be evaluated.

## 2 Materials

**Figure 1:**
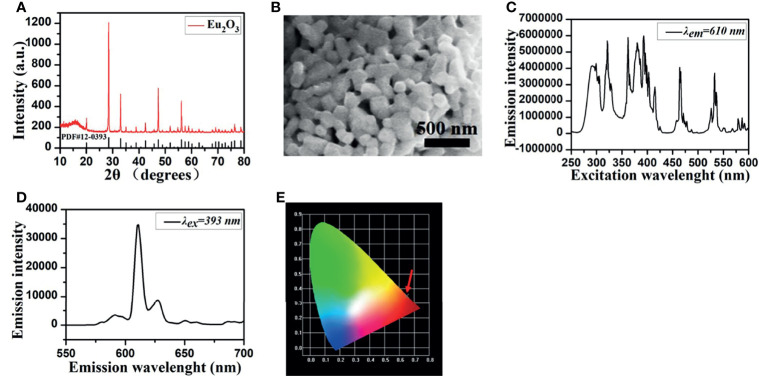
EO nanoparticle morphology and characterization. **(A)** XRD patterns of EO. **(B)** SEM visualization. **(C)** The optical excitation spectrum with 610 nm exhibits the characteristic absorption peaks at 292, 321, 362, 380, 393, 464, and 533 nm. **(D)** The emission spectra of EO excited by 393 nm. **(E)** CIE chromaticity diagram of EO.

As X-ray excitable nanophosphors, europium oxide (Eu_2_O_3_, EO) (Shanghai Aladdin Biochemical Technology Co., Ltd., China, CAS No. 1308-96-9) was used in our research, and the structure and characterization of EO nanoparticles are shown in **Figure 1**. The crystal structure of EO was examined by using X-ray diffraction, and the structure of cubic phase europium oxide is presented in **Figure 1A**. The microstructures of EO were explored *via* field emission scanning electron microscopy. The result shows that EO nanoparticles are with peanut-like morphology (**Figure 1B**). The luminescence properties of Eu^3+^-based nanometer materials are based on the mechanism of emission of Eu^3+^. When EO is excited by fluorescence or X-ray, it should exhibit similar luminous properties. In order to get emission wavelengths of EO in our experiments, photoluminescence (PL) properties were assayed on a luminescence spectrometer (HORIBA, Model FluoroMax-4p, USA) with a xenon discharge lamp at room temperature. We measured to figure out the emission wavelengths of EO as shown in **Figure 1D**, the highest luminescence peak locates at 610 nm and the second high luminescence peak locates at 630 nm, and the corresponding suitable excitation spectra of 393 nm are shown in **Figure 1C**. This conclusion is similar with the study of Hu et al. which investigated fluorescence characterization of EO (35). The Commission international de l’Eclairage (CIE) coordinates served as a tool to figure out the optical mechanism of the human eye exposed to a specified spectrum (21). The calculated chromatic coordinates of EO powders are (0.6490, 0.3506), as shown in **Figure 1E**. From the CIE chromaticity diagram, the luminescence area of EO is located at the red region apparently.

EO phosphor is suitable to be a luminescent substance in living organisms for XLCT contributes from their good penetration performance of the emission light, and 610 and 630 nm are chosen as the emission wavelengths of the simulation experiments because of the desirable amount of emission intensity under the two wavelengths.

## 3 Methods

### 3.1 Mathematical Model of X-Ray Transmission in Biological Tissues

In the XLCT imaging system, photons are produced due to stimulated radiation that can be described as follows (13):


(1) S(r)=ϵX(r)ρ(r)

where *S*(*r*) is the light source, *ϵ* is the luminescence yield of the nanophosphor target, *X*(*r*) is the X-ray intensity at position *r*, and *ρ*(*r*) is the nanophosphor density at position *r*.

According to Lambert–Beers’ law, the energy distribution of X-ray transmitted in biological tissues can be expressed as (13):


(2) $$X(r)=X_{0}\exp\{-\int_{r_{0}}^{r}\mu_{t}(\tau )d\tau \}$$

where *μ_t_* (*τ*) is the X-ray attenuation coefficient at positon *τ* which can be obtained *via* the CT technique.

### 3.2 Mathematical Model of Light Transmission in Biological Tissues

In a hybrid model, according to the optical parameter, the non-homogeneous solution domain is divided into the DE model applicable region (Region1) and the *SP*_3_ model applicable region (Region2).

**Figure 2:**
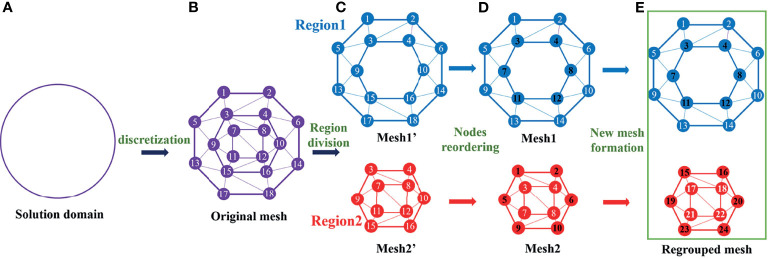
The mesh regrouping process in simplified 2D formation.

The three-dimensional solution domain can be discretized into a tetrahedron mesh. For convenient representation, we first explain in 2D form, the 2D circular solution domain (**Figure 2A**) was discretized into a triangular mesh (**Figure 2B**). The grid information is represented as:


(3) $$V=\{N_{i},T_{j}\},i=1,2,\ldots n,j=1,2,\ldots t.$$

Where *N_i_* = (*x_i_*,*y_i_*), (*x_i_*,*y_i_*) is the coordinate of the *i*th node and *n* is the number of nodes. *T_j_* = (*a_j_*,*b_j_*,*c_j_*) stores the information about triangles, whose number is *t*, and (*a_j_*,*b_j_*,*c_j_*) are the ordinal number of the three vertices of the *j*th triangle. The connection between the triangle units and the nodes is established, and nodes are arranged according to their spatial location in this discrete mesh.

After region division, Region1 adopts the DE approximate modeling to get the matrix equation (36):


(4) $$M_{ij}\Phi =F_{ij}S$$

Where


(5) $$M_{i,j}=\int_{\Omega }(D(r)(\nabla \varphi_{j}(r))(\nabla \varphi_{k}(r))+\mu_{a}\varphi_{j}(r)\varphi_{k}(r))dr+\int_{\partial \Omega }\frac{\varphi_{j}(r)\varphi_{k}(r)}{2A_{n}(r)}dr$$

(6) $$F_{i,j}=\int_{\Omega }\varphi_{j}(r)\varphi_{k}(r)dr$$

Ω is the region of interest, Φ is the light density, *S* is the power density of the light source, *D*(*r*) = 1/(3(*μ_a_* + (1 − *g*)*μ_s_*)) is the optical diffusion coefficient, *μ_a_* is the absorption coefficient, *μ_s_* is the scattering coefficient, *g* is the anisotropy parameter, and *A_n_* is the refractive mismatch factor at the boundary ∂Ω.

Region2 uses *SP*_3_ approximate modeling to get the matrix form:


(7) $$\left[\begin{array}{ll} M_{1\Phi_{1}} & M_{1\Phi_{2}}\\ M_{2\Phi_{1}} & M_{2\Phi_{2}} \end{array}\right]\left\{\begin{array}{c} \phi_{1}\\ \phi_{2} \end{array}\right\}=\left[\begin{array}{ll} F_{11} & 0\\ 0 & F_{22} \end{array}\right]\left\{\begin{array}{c} S\\ \frac{-2}{3}S \end{array}\right\}$$

where the corresponding components in the block matrixes denote (31):


(8) $$\{\begin{array}{l} M_{1\Phi_{1}{}^{jk}}=\int_{\Omega }\left(\frac{1}{3\mu_{a1}}\right)\nabla v_{j}(r)\nabla v_{k}(r)+\mu_{a}v_{j}(r)v_{k}(r)dr-\int_{\partial \Omega }\frac{\xi_{11}}{3\mu_{a1}}v_{j}(r)v_{k}(r)dr\\ M_{1\Phi_{2}{}^{jk}}=-\int_{\partial \Omega }\frac{\xi_{12}}{3\mu_{a1}}v_{j}(r)v_{k}(r)dr\\ M_{2\Phi_{1}{}^{jk}}=-\int_{\Omega }\left(\frac{2\mu_{a}}{3}\right)v_{j}(r)v_{k}(r)dr-\int_{\partial \Omega }\frac{\xi_{21}}{7\mu_{a3}}v_{j}(r)v_{k}(r)dr\\ M_{2\Phi_{2}{}^{jk}}=\int_{\Omega }\left(\frac{1}{7\mu_{a3}}\right)\nabla v_{j}(r)\nabla v_{k}(r)+(\frac{4}{9}\mu_{a}+\frac{5}{9}\mu_{a2})v_{j}(r)v_{k}(r)dr-\int_{\partial \Omega }\frac{\xi_{22}}{7\mu_{a3}}v_{j}(r)v_{k}(r)dr \end{array}$$

(9) $$F_{11jk}=F_{22jk}=\int_{\Omega }v_{j}(r)v_{k}(r)dr$$

*ξ_s_*,*_t_* (*s*, *t* = 1,2) are the boundary coefficients and are calculated based on (37).

Equations (5) and (6) are for the nodes that belong to Region1 and Equations (8) and (9) are for the nodes that belong to Region2. System matrix *M*_2_ consists of four components, whose dimension is different from *M*_1_. These matrixes corresponding to Region1 and Region2 should be built separately, so discrete nodes and tetrahedrons should be classified according to their regions to support the calculation of the corresponding model. In Mesh1′ and Mesh2′ (**Figure 2C**), the classification of nodes and tetrahedrons destroys their original structure based on spatial position, because the region division principle is based on the optical properties of the regions rather than the spatial position.

To ensure the continuity of nodes and tetrahedrons in each region, the nodes are reordered according to their respective regions in **Figure 2D**, and the information of new meshes is represented as:


(10) $$\{\begin{array}{l} V_{1}=\{N_{i1},T_{j1}\},\;i1=1,2,\ldots ,n_{1},j1=1,2\ldots ,t_{1},\\ V_{2}=\{N_{i2},T_{j2}\},i2=1,2,\ldots ,n_{2},j2=1,2\ldots ,t_{2}. \end{array}$$

The nodes in both meshes are sorted by their current positions, and the triangles are formed by regarding existing nodes as vertices. Comparing the spatial positions of the nodes in the two meshes, these nodes sharing the same spatial location (black number nodes in **Figure 2D**) satisfy:


(11) $$N_{i1}\subseteq V_{1}=N_{i2}\subseteq V_{2}$$

In order to handle this hybrid problem, Mesh1 and Mesh2 are combined into a whole regrouping mesh as shown in **Figure 2E**, and the regrouped mesh information is represented as:


(12) $$V\prime =\left\{\left[\begin{array}{c} N_{i1}\\ N_{i2} \end{array}\right],\left[\begin{array}{c} T_{j1}\\ T_{j2} \end{array}\right]\right\}\{\begin{array}{l} i1=1,2,...,n_{1},i2=n_{1}+1,n_{1}+2,...,n_{1}+n_{2},\\ j1=1,2,...,t_{1},j2=t_{1}+1,t_{1}+2,...,t. \end{array}$$

where *n*_1_ and *n*_2_ are the number of nodes in Mesh1 and Mesh2.

In the process of dividing and regrouping mesh, the boundary elements extracting only consider the initial outer boundary of the solution domain, and the boundary nodes are also reordered in the regrouping mesh.

The two meshes meet at a boundary $\partial \Omega_{DE}/\Omega_{SP_{3}}$ and the luminous flux at the boundary must remain continuous. It should be guaranteed that the boundary nodes meet the following condition:


(13) $$\varphi_{DE}=\varphi_{SP_{3}}$$

In the process of solving the hybrid model, the system matrix, source weight matrix, and power density of light source obtained by DE and *SP*_3_ models need to be united.

Equation (4) can be transformed as


(14) $$M_{1}\Phi_{1}=F_{1}S_{1}$$

and Equation (7) can be transformed as


(15) $$M_{2}\Phi_{2}=F_{2}S_{2}$$

**Figure 3:**
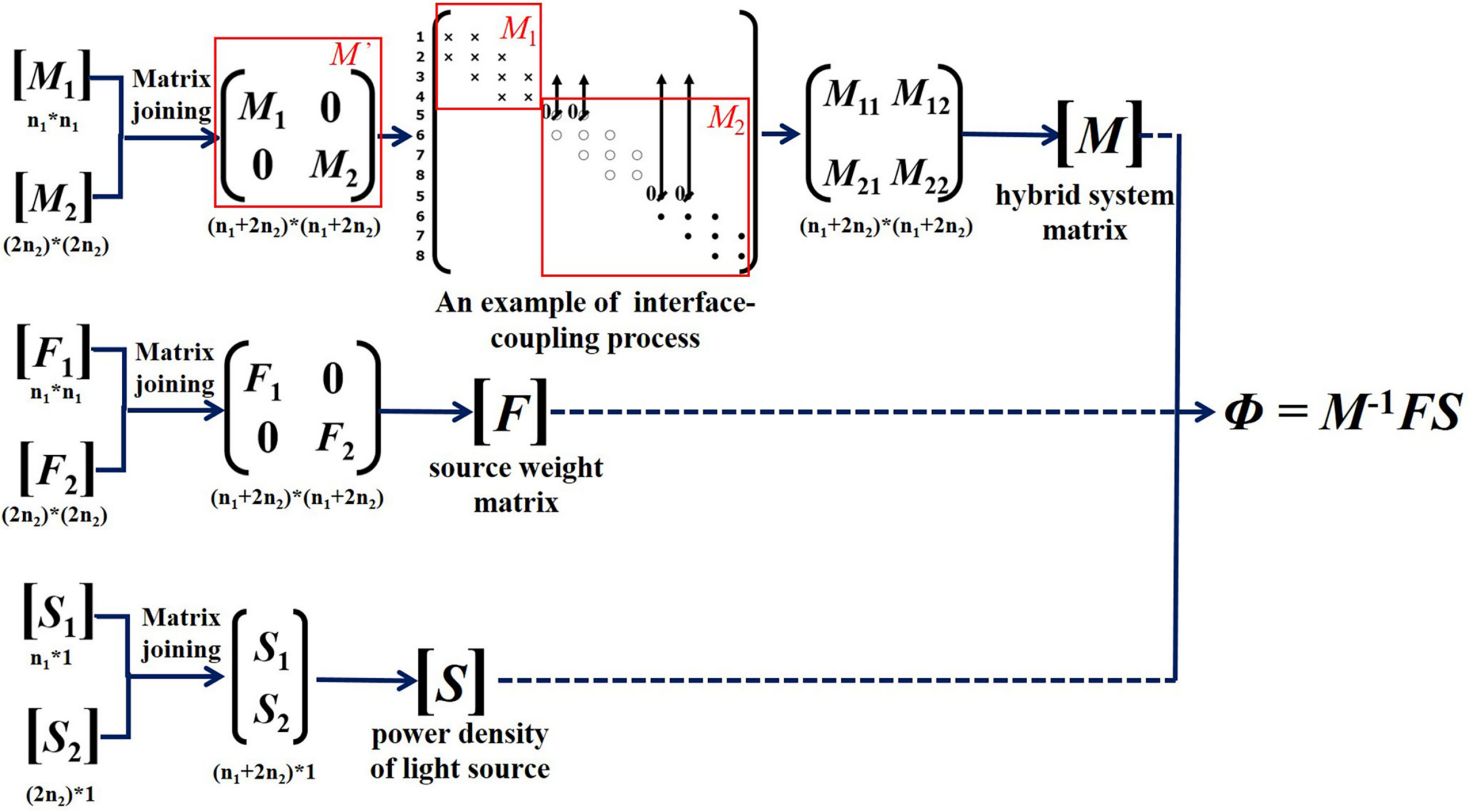
Hybrid transport model construction.

The merging operation of the corresponding matrixes is shown in **Figure 3**, and the joining process of system matrix *M* is relatively complex.

The nodes on the boundary $\partial \Omega_{DE}/\Omega_{SP_{3}}$ locate at the same point in space, and duplicates are generated during the formation of the hybrid system matrix. The duplicate terms of the system matrix *M'* from the *M*_1_ and *M*_2_ parts are shown in **Figure 3**, and one row of the *M*_1_ part (fork-marked elements) corresponds to two rows of the *M*_2_ part (circle- and point-marked elements). In order to avoid the repeated contributions of boundary nodes, these nodes need to be coupled (28).

According to the boundary conditions, Equation (13), and DE and *SP*_3_ model theories, *φ*, *φ*_1_, and *φ*_2_, which are components of surface light fluence, meet the following requirements (28):


(16) $$\varphi_{1}=-\varphi +\frac{2}{3}\varphi_{2},\varphi_{2}=\frac{3}{2}\varphi -\frac{3}{2}\varphi_{1}$$

The corresponding elements of the system matrix *M'* must change accordingly, and it has the form shown in **Figure 3**, which marks each row with its corresponding node number. For example, the entries for node 3 and node 5 locate at the same point in space, so the fields should be coupled. The matrix entries indicating node 5 are moved to row 3 of the system matrix and then each row indicating node 5 is set as zero. The diagonal elements indicating node 5 are then set as 1 to reestablish its relationship with itself. The relationship with the other field fluence is then established by Equation (16) (28). Through the process above, the corresponding items in the hybrid system matrix *M'* are operated, which could be represented by the block matrix *M*_11_, *M*_12_, *M*_21_, and *M*_22_, and the coupled hybrid system matrix *M* is obtained after repeated term coupling.

In the whole regrouping mesh, the relationship between the photon flux density on the surface and the power density of the light source is established:


(17) MΦ=FS

It can be transformed as:


(18) Φ=BS

with *B* = *M* ^-1^ *F*. The rows of matrix *B* that correspond to the row number of unmeasurable photon fluence rate Φ*^μ^* are eliminated, which can be represented as a set of linear equations of the form:


(19) $$A_{\lambda }S=\Phi_{\lambda }^{m}$$

where *A* is a sensitivity matrix at a given wavelength *λ*, and Φ*^m^* is the measurable photon fluence rate (on the surface) at the same wavelength. As the imaging problem is known to be non-unique, it has been shown that measuring at multiple wavelengths can help overcome this issue caused by the unique spectrally varying attenuation of biological tissue (38). Assuming there are two wavelengths *λ* in Eq. (19), Eq. (20) is deduced:


(20) $$\left[{}_{A_{\lambda 2}}^{A_{\lambda 1}}\right]S=\left[{}_{\Phi_{\lambda 2}{}^{m}}^{\Phi_{\lambda 1}{}^{m}}\right]$$

The output-least-squares formulation containing a regularization term is used, and the solution can be determined by minimizing the following energy function:


(21) $$\min\frac{1}{2}{\left||AS-\Phi^{m}|\right|}_{2}^{2}+\tau \left||S|\right|$$

*τ* > 0 is a regularization parameter, and the incomplete variables truncated conjugate gradient algorithms (6) is used to solve this problem.

## 4 Experimental Design

In this section, numerical simulations and *in-vivo* experiments were designed to evaluate the performance of the finite element mesh regrouping strategy-based hybrid light transport model in XLCT. All programs were run on a computer with an Intel(R)Core(TM)i7 – 6700CPU (3.40 GHz) and 16 – GB RAM.

### 4.1 Numerical Simulation Setup

**Figure 4:**
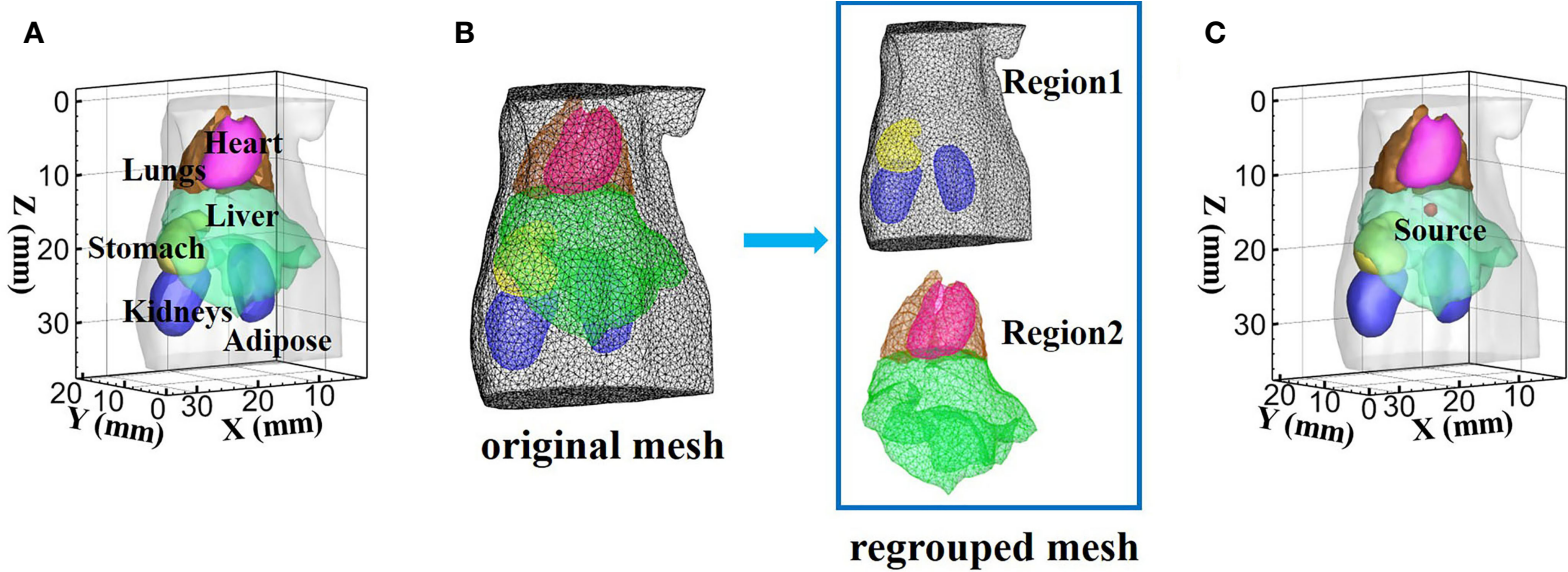
The digital mouse used in the simulation experiment. **(A)** The mouse model with six organs. **(B)** Display of mesh regrouping in a mouse model. **(C)** Model of source in the liver.

The commonly used digital mouse model was employed to forward simulation and reconstruction for XLCT, and only the torso section of the mouse with a height of 35 mm was selected as the region to be investigated, including adipose, heart, liver, lungs, stomach, and kidneys (**Figure 4A**). At the wavelength of 610 and 630 nm, the absorption coefficient *μ_a_*, the scattering coefficient *μ_s_*, and the anisotropy coefficient *g* of these tissues are listed in **Table 1**. The optical properties were calculated using the formula summarized in (39). According to the optical parameters in **Table 1** and the conclusions of (40), at the wavelength of 610 nm, the heart, liver, and lungs are suitable to adopt the *SP*_3_ approximate modeling, while in adipose, stomach, and kidneys, a similar performance can be achieved whether the *SP*_3_ or DE approximate model is adopted. Thus, adipose, stomach, and kidneys adopted the DE model for lower computational complexity. At 630 nm wavelength, the same classification was obtained. Therefore, adipose, stomach, and kidneys belonged to Region1, while heart, liver, and lungs belonged to Region2 in the experiments of this paper. The mouse model was discretized by the finite element method, and the new regrouped mesh was formed according to the division of the two regions (**Figure 4B**). A spherical source with 1 mm radius was placed in the liver and its center locates at (19 mm; 8 mm; 14.5 mm) as shown in **Figure 4C**. The forward mouse model was discretized into 117,260 tetrahedral elements and 22,155 nodes, while the inverse mouse model was discretized into 55,215 tetrahedral elements and 10,801 nodes.

**Table 1:** Optical parameters of the mouse tissues for 610 and 630 nm.

Tissue	610 nm			630 nm		
	*μ_a_* (mm^–1^)	*μ_s_* (mm^–1^)	g	*μ_a_* (mm^–1^)	*μ_s_* (mm^–1^)	g
Adipose	0.0127	21.1547	0.94	0.0069	20.7961	0.94
Heart	0.2015	7.3484	0.85	0.1085	7.0171	0.85
Stomach	0.0384	19.6728	0.92	0.0207	19.0667	0.92
Liver	1.2086	7.4826	0.90	0.6505	7.2334	0.90
Kidneys	0.2258	18.5421	0.86	0.1216	17.6605	0.86
Lungs	0.6687	38.0785	0.94	0.3622	37.4330	0.94

### 4.2 *In-Vivo* Experiment Setup

The application potential of the proposed MRHM-based method was then demonstrated by a living mouse-based *in*-*vivo* experiment.

The XLCT/micro-CT dual-mode system developed by our laboratory was used to collect data. The XLCT system consists of a micro-focus cone beam X-ray source (L9181-02, Japan); a highly sensitive electron-multiplying charge coupled device (EMCCD) camera (iXon Ultra, Andor, Northern Ireland), which is coupled with a 24 mm f/1.4L lens (Canon, Japan) for optical imaging; and an X-ray flat-panel detector (C7942CA-22, Japan) for high-resolution CT imaging.

All animal experiments were conducted under the approval of the Animal Ethics Committee of the Northwest University of China. A female BALB/c nude mouse (6–8 weeks old) was used to establish a source-implanted mouse model. After the mouse was anesthetized with pentobarbital (50 mg/kg, 0.1 ml, IP injection), a transversal incision was made in the abdomen. Then, the liver lobe was gently lifted, and a plastic tube with a diameter of 1 mm and a height of 5 mm filled with about 20 μl nanomaterial luminescent material EO of concentrations 200 mg/ml was implanted in the abdomen of the nude mouse. About 3 min later, the mouse was used for luminescence imaging.

In the luminescence image acquisition process, single projection data were obtained with a 120 field of view (FOV). The EMCCD camera coupled with 10 nm FWHM bandpass filters centered at 610 nm (Thorlabs FB610-10) was adopted to acquire the optical images. The exposure times, the EM gain, and image binning were set to 60 s, 1, and 1 × 1. After obtaining the optical measurement data, the mouse was kept motionless and scanned by micro-CT. In the X-ray scanning progress, the voltage and power were set to 90 kV and 27 W, respectively. A total of 600 X-ray projections were obtained with an interval of 0.6 degree and each projection integrating time of 0.5 s. The CT data of the mouse were reconstructed using the GPU-accelerated Feldkamp–Davis–Kress (FDK) algorithm.

### 4.3 Quantitative Evaluation

In order to validate the advantages of MRHM, in forward simulation, the surface light flux calculated by the Monte Carlo method (MC) was taken as the standard for comparison, and DE, *SP*_3_, and the hybrid diffusion equation–*SP_N_* method (HDSM) which was presented in (33) served as the optical transmission models for comparative experiments.

Average relative error (ARE) is described as a quantitative evaluation index in forward simulation (33, 40), which is the average relative error of the calculated results of the DE, *SP*_3_, HDSM, or MRHM and the simulated one of MC on the surface detection points. Its calculation method follows:


(22) $$ARE=\frac{\Sigma_{i=1}^{N}(abs(Simulation_{i}-MC_{i})/\max(MC_{i}))}{N}$$

where *Simulation_i_* is the surface energy value obtained by MATLAB simulation and *MC_i_* is the surface energy value obtained by MC, and *N* is the total number of sample points. The smaller the *ARE* values, the better the performance of the calculated method.

The *t*1 and *t*2 (in units of seconds) record the construction time of the system matrix and the time of inverse operation in forward simulation, respectively.

To verify the feasibility and applicability of MRHM in source reconstruction, several common indicators were used: location error (LE), Dice, and CNR were used to evaluate the target location, shape recovery, and image contrast of the adopted methods, respectively. These indicators can be calculated as follows:


(23) $$LE=\sqrt{{\left(x-x_{0}\right)}^{2}+{\left(y-y_{0}\right)}^{2}+{\left(z-z_{0}\right)}^{2}}$$

where (*x*, *y*, *z*) and (*x*_0_,*y*_0_,*z*_0_) are the coordinates of reconstruction energy weighted center point and the real source center, respectively.


(24) $$Dice=\frac{2\left|X\cap Y\right|}{\left|X\right|+\left|Y\right|}$$

where *X* and *Y* denote the regions of the reconstructed and actual sources, respectively.


(25) $$CNR=\frac{\left|\mu_{ROI}-\mu_{BCK}\right|}{{\left(\omega_{ROI}\sigma^{2}{}_{ROI}+\omega_{BCK}\sigma^{2}{}_{BCK}\right)}^{1/2}}$$

where the subscripts *ROI* and *BCK* denote the target and background regions of the imaged object: the *ROI* corresponds to the nodes within the reconstructed light source, and *BCK* corresponds to the remaining nodes; *μ*, *w*, and *σ* represent the average intensity value, weighting factor, and variance, respectively.

In the process of source reconstruction, the construction time of sensitivity matrix *A* is represented by *T* (in units of seconds).

## 5 Results

### 5.1 Numerical Simulations

#### 5.1.1 Forward Simulation

Optical transmission models including DE, *SP*_3_, HDSM, and MRHM were used for forward simulation; in the hybrid optical transmission model HDSM and MRHM, the same tissue classification was performed according to the experimental setup.

**Figure 5:**
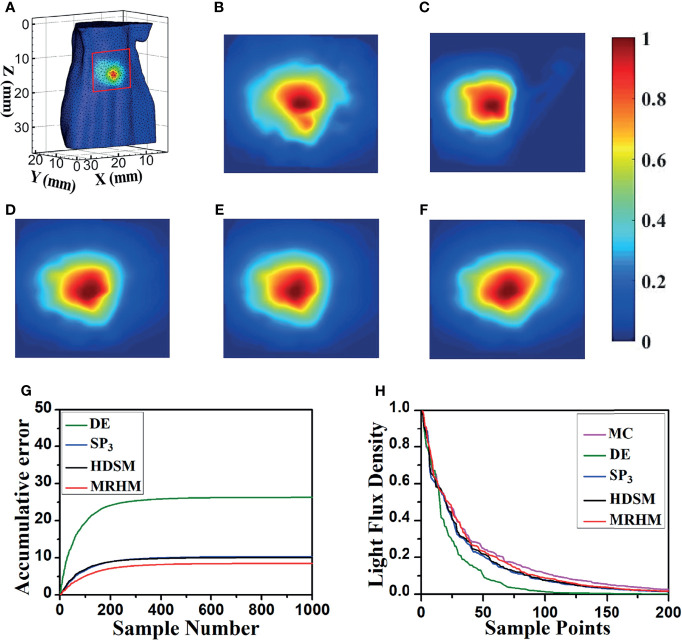
Results of forward simulation at 610 nm. **(A)** The surface luminescence flux illustration of the digital mouse model. **(B–F)** The surface luminescence fluxes projected onto the *X*–*Z* plane simulated by the MC, DE, *SP*_3_, HDSM, and MRHM, respectively. **(G)** Accumulative error for each model. **(H)** The descending order of surface light flux of each model.

**Figure 5A** shows the surface luminescence flux illustration of the digital mouse model at 610 nm. **Figures 5B–F** show the *X*–*Z* plane projections of the surface light flux calculated by MC, DE, *SP*_3_, HDSM, and MRHM under 610 nm emission wavelength, respectively. For a better comparison, all of the results were exhibited in the same range of surface energy value. Compared with the result of MC in **Figure 5B**, the light distribution in **Figure 5C** is significantly different, and the light distribution in **Figures 5D–F** is all similar to **Figure 5B**.

Furthermore, to reflect the experimental results accurately and intuitively, 1,000 highest-energy-value surface nodes were selected from the surface light flux distribution calculated by MC, and the values of surface energy at these nodes acquired by MC, DE, *SP*_3_, HDSM, and MRHM were used for calculation and comparison. Firstly, the ARE of each model was calculated and shown in **Table 2**, and MRHM has the minimum ARE. Then, the surface light flux distributions obtained by each model were subtracted with the result of MC at the corresponding node; The accumulative errors with the increasement of nodes number are shown in **Figure 5G**. The accumulative error is consistent with the ARE, and the error of MRHM is the minimum. The accumulative error curves of *SP*_3_ and HDSM models are very close with a minor difference, which is consistent with the ARE in **Table 2**. In addition, to further verify the energy distribution differences of each model, **Figure 5H** shows the surface light flux of each model in descending order, which embodies that *SP*_3_ and HDSM models are also close, and the curves of MRHM and MC are relatively close.

**Table 2:** Quantitative results in forward simulations.

Wavelength	Model	ARE	System matrix dimension	*t*_1_ (s)	*t*_2_ (s)
610	DE	0.02627	22,155*22,155	68.38	71.17
	*SP*_3_	0.01028	44,310*44,310	1,639.36	81.16
	HDSM	0.01007	44,310*44,310	248.77	428.43
	MRHM	0.00850	30,610*30,610	272.83	56.15
630	DE	0.00674	22,155*22,155	66.11	65.21
	*SP*_3_	0.00649	44,310*44,310	1,749.22	79.28
	HDSM	0.00667	44,310*44,310	226.57	381.06
	MRHM	0.00622	30,610*30,610	273.23	57.68

The time-consuming comparison results of this set of experiments are shown in **Table 2**. Since the system matrix dimensions of *SP*_3_ and HDSM are twice that of DE, they take longer computation time. MRHM was used to reduce the system matrix dimension, which saved 83.4% of the system matrix construction time (*t*1) compared with *SP*_3_ and reduced 86.9% of the inverse calculation time (*t*2) compared with HDSM.

**Figure 6:**
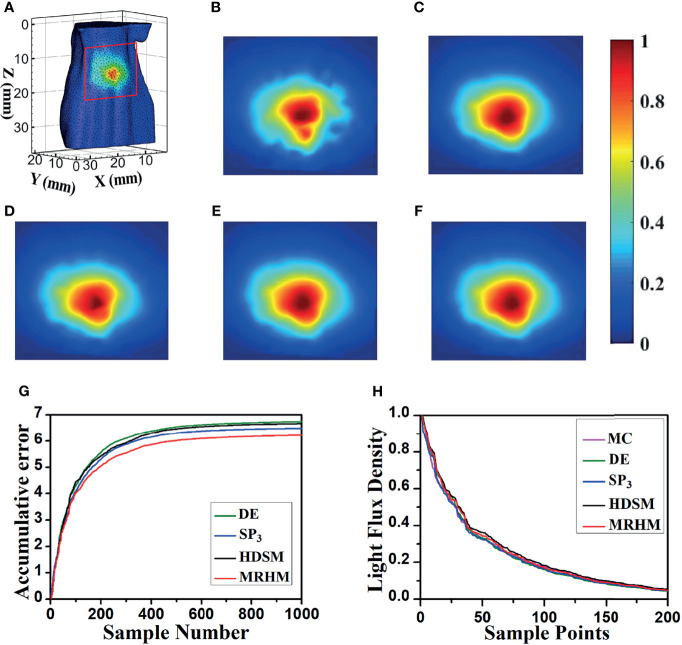
Results of forward simulation at 630 nm. **(A)** The surface luminescence flux illustration of the digital mouse model. **(B–F)** The surface luminescence fluxes projected onto the *X*–*Z* plane simulated by the MC, DE, *SP*_3_, HDSM, and MRHM, respectively. **(G)** Accumulative error for each model. **(H)** The descending order of surface light flux of each model.

To verify the applicability of the proposed model, another emission wavelength of EO, 630 nm, was used in the experiment. Similar to the experimental setup at 610 nm, **Figure 6A** shows the surface luminescence flux illustration of the digital mouse model at 630 nm, and the *X*–*Z* plane projections of the surface light flux calculated by MC, DE, *SP*_3_, HDSM, and MRHM are shown in **Figures 6B–F**, and the light distributions in **Figures 6C–F** are similar to **Figure 6B**, which is the light distribution of MC. The ARE in **Table 2** indicates that the error of MRHM is less than that of the other models, and the accumulative errors (**Figure 6G**) come to the same conclusion. The descending order of the surface light flux of each model is shown in **Figure 6H**, and the characteristics of each curve are relatively similar and are all close to that of MC, which indicates that all these models have high veracity. The results of ARE and accumulative error show that MRHM has the highest accuracy among the four models.

Simultaneously, MRHM has more advantages in time cost (**Table 2**) in this set of experiments, which saved 84.4% of the system matrix construction time (*t*1) compared with *SP*_3_ and reduced 84.9% of the inverse calculation time (*t*2) compared with HDSM.

According to optical parameters corresponding to 610 nm wavelength, the liver is a low-scattering high-absorption organ, and the DE model in this case is not a proper choice, which leads to the largest simulation error compared with the other models. Thus, in **Figure 5**, the surface light distributions of the DE model are obviously different from that of MC with the largest accumulative error, while the absorption coefficient of the liver is reduced in half at 630 nm than at 610 nm. In this case, the performance of the DE model has a great improvement. Thus, the surface light distributions of the DE model are similar with those of MC, the corresponding accumulative error of DE is relatively reduced and slightly larger than that of other models.

#### 5.1.2 Inverse Simulation

To verify the feasibility and applicability of the proposed method in the reconstruction of light source, inverse simulation was performed. To ensure accuracy and efficiency, these surface measurements for reconstruction were calculated using MC, and the light transport model of reverse transmission adopted DE, *SP*_3_, HDSM, and MRHM, respectively. The emission wavelengths and the division of tissue regions correspond to their respective forward simulation.

**Figure 7:**
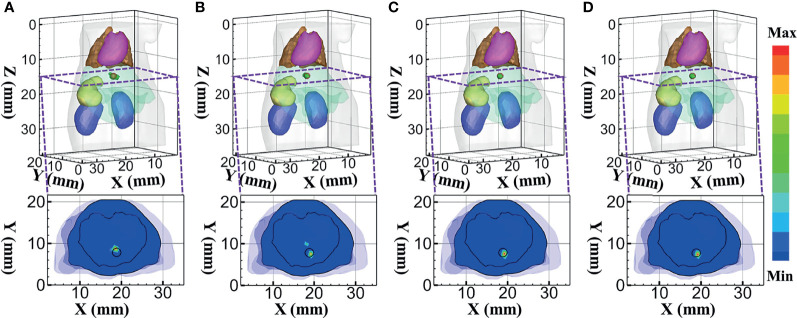
Reconstructed results at 610 nm. **(A–D)** 3D views of the reconstructed results and the corresponding sectional images obtained by the DE-, *SP*_3_-, HDSM-, and MRHM-based methods, respectively.

Firstly, the reconstruction results obtained using DE, *SP*_3_, HDSM, and MRHM corresponding to 610 nm emission wavelength are shown in **Figure 7**. **Figures 7A–D** show the 3D views of the reconstructed results and their sectional images (*Z* = 14.5 mm) of each model-based reconstruction method. The red spherical in the 3D views and the black circle in the sectional images label the actual position of the real sources, while the green irregular shapes are the reconstructed sources. The results show that the reconstructed images using *SP*_3_-, HDSM-, and MRHM-based reconstruction methods have almost the same quality and better than the reconstruction quality of the DE-based reconstruction method. In addition, the DE- and *SP*_3_-based methods result in an artifact around the source, and HDSM and MRHM can achieve satisfactory results.

To quantitatively evaluate these images, we calculated the indicators of LE, Dice, and CNR. Those indicators obtained under each model-based method are shown in **Table 3**. The *SP*_3_-, HDSM-, and MRHM-based methods have similar LE value of about 0.5 mm, which is much smaller than that of the DE-based method (1.013 mm), and the LE of MRHM is the minimum. The Dice draws a similar conclusion; the reconstruction region of *SP*_3_-, HDSM-, and MRHM-based methods is more similar to the real source than that of the DE-based method, and the CNR of the MRHM-based method is the largest among the four methods. These results indicate that the MRHM-based method performs better in target location, shape recovery, and image contrast, compared with the other methods in this set of experiments. The construction time *T* of the sensitivity matrix indicates that MRHM saves 70.5% of the computation time compared with *SP*_3_ and reduces 96.4% of that compared with HDSM.

**Table 3:** Quantitative results in reconstruction experiments.

Wavelength	Model	Real source center (mm)	Reconstructed source center (mm)	LE (mm)	Dice	CNR	*T* (*s*)
610	DE	(19, 8, 14.5)	(18.32, 8.72, 14.68)	1.013	0.38	1.110	36.07
	*SP*_3_		(19.18, 7.55, 14.37)	0.502	0.53	3.338	1,665.19
	HDSM		(19.17, 7.55, 14.38)	0.496	0.53	3.482	13,483.47
	MRHM		(19.16, 7.58, 14.37)	0.465	0.53	8.268	491.43
630	DE	(19, 8, 14.5)	(18.09, 6.95, 13.68)	1.618	0.43	0.659	37.97
	*SP*_3_		(18.19, 7.87, 14.10)	0.910	0.53	2.623	1,658.26
	HDSM		(18.18, 7.85, 14.09)	0.934	0.53	2.556	13,468.56
	MRHM		(18.57, 8.85, 14.46)	0.719	0.71	3.525	478.97

**Figure 8:**
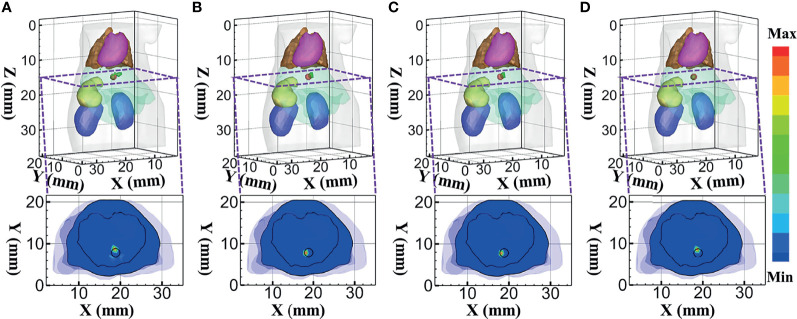
Reconstructed results at 630 nm. **(A–D)** 3D views of the reconstructed results and the corresponding sectional images obtained by the DE-, *SP*_3_-, HDSM-, and MRHM-based methods, respectively.

At 630 nm emission wavelength, the 3D views of the reconstructed results and their sectional images (*Z* = 14.5 mm) of each model-based reconstruction method are shown in **Figures 8A–D**. From the results, the DE-based method results in an artifact around the source, and the reconstructed images using the MRHM-based reconstruction method are the best compared with the other three methods.

Combining the quantitative results in **Table 3** with the minimum LE and the maximum Dice and CNR indicates that the MRHM-based reconstruction method also performs better in target location, shape recovery, and image contrast, when compared with the other light transport model-based methods corresponding to 630 nm emission wavelength. Simultaneously, MRHM has more advantages in time cost (**Table 3**) in this set of experiments, which saves 71.1% of the sensitivity matrix construction time compared with *SP*_3_ and reduces 96.4% of that compared with HDSM.

**Figure 9:**
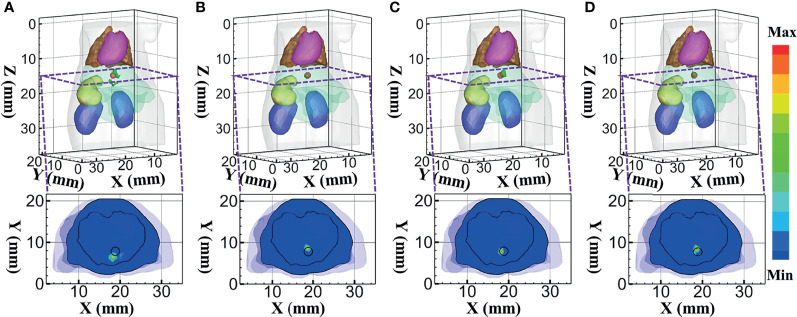
Reconstructed results at multispectral. **(A–D)** 3D views of the reconstructed results and corresponding sectional images obtained by the DE-, *SP*_3_-, HDSM-, and MRHM-based methods, respectively.

To further verify the performance of the MRHM-based method, the multispectral experiment was carried out for the source reconstruction. These surface measurements for reconstruction were calculated using MC at 610 and 630 nm emission wavelengths. The reconstruction results corresponding to the multispectral experiments are shown in **Figure 9**. **Figures 9A–D** show the 3D views of the reconstructed results of each model-based reconstruction method and their sectional images (*Z* = 14.5 mm). From the results, the DE-based method shows a big deviation from the source and results in an artifact around the source, and the 3D reconstructed image and sectional image using the MRHM-based reconstruction method are the best compared with those using the other three methods.

According to the quantitative results in **Table 4**, LE of the MRHM-based reconstruction method is minimum, whose Dice and CNR are maximum. The MRHM-based reconstruction method also has good performance in multispectral source reconstruction. The time-consuming comparison results of this set of experiments are shown in **Table 4**. As the dimension of the sensitivity matrix increases in multispectral experiments, the advantage of MRHM in computation time becomes apparent, which saves 2,386.92 s (71.5%) of the sensitivity matrix construction time compared with *SP*_3_ and reduces 25,974.16 s (96.5%) of that compared with HDSM.

**Table 4:** Quantitative results in multispectral reconstruction experiment.

Wavelength	Model	Real source center (mm)	Reconstructed source center (mm)	LE (mm)	Dice	CNR	*T* (*s*)
Multispectral	DE	(19, 8, 14.5)	(18.57, 6.94, 13.99)	1.257	0.33	0.730	70.85
	*SP*_3_		(18.63, 8.81, 14.62)	0.897	0.53	3.602	3,340.19
	HDSM		(18.20, 7.89, 14.11)	0.898	0.53	2.672	26,927.43
	MRHM		(18.61, 8.45, 14.19)	0.672	0.80	5.979	953.27

These inverse simulations draw similar conclusions as the forward simulations. The MRHM-based method has well recovered the position and distribution of the true source and achieves a better balance between accuracy and efficiency than the other model-based methods, and it is indeed an optimal option as a light transport model for XLCT.

### 5.2 *In-Vivo* Experiments

**Figure 10:**
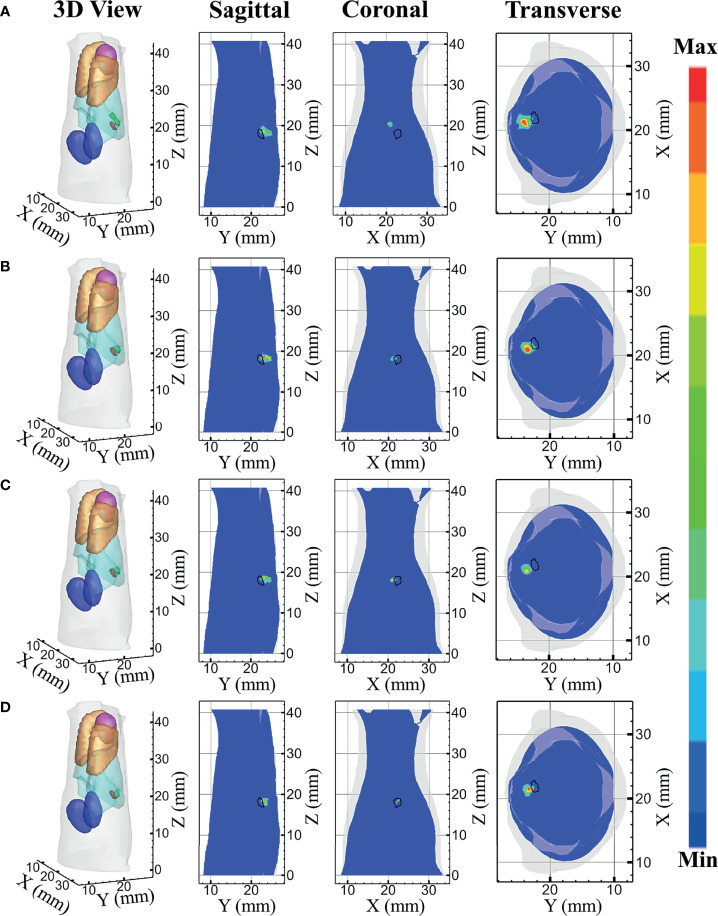
Reconstruction results of *in-vivo* experiments. **(A–D)** The 3D view, sagittal view, coronal view, and transverse view of the reconstructed results obtained by the DE-, *SP*_3_-, HDSM-, and MRHM-based methods, respectively.

The reconstructed results of the *in-vivo* experiments performed by each model-based reconstruction method are shown in **Figure 10**. **Figures 10A–D** represent the DE, *SP*_3_, HDSM, and MRHM model-based methods, respectively. The 3D views of the reconstructed results are displayed in the first column, and the real source and reconstructed source positions are represented by red regions and green irregular shapes, respectively. Sagittal, coronal, and transverse planes are determined according to the central position of the true source as shown in the next sequence, and the irregular shape black circle in the sectional images labels the actual position of the real source. The DE-based method leads to a big deviation from the source, and the shape of the reconstructed source is larger, which results in an artifact around the source. The reconstructed images of *SP*_3_ and HDSM are similar, which are not as good as the reconstructed result of MRHM. The reconstructed source location of the MRHM-based method is the closest to the real source in sagittal, coronal, and transverse plane images.

The quantitative analysis of the reconstructed source is recorded in **Table 5**. The MRHM-based reconstruction method has the minimum LE and the maximum Dice and CNR, and it performs better in target location, shape recovery, and image contrast, compared with DE, *SP*_3_, and HDSM. Simultaneously, MRHM has more advantages in time cost, which saves 76.8% of the sensitivity matrix construction time compared with *SP*_3_ and reduces 95.4% of that compared with HDSM.

**Table 5:** Quantitative results of the *in-vivo* experiment.

Model	LE (mm)	Dice	CNR	*T* (s)
DE	2.479	0	3.121	178.78
*SP*_3_	1.848	0.12	3.678	2,054.61
HDSM	1.845	0.13	3.716	10,306.12
MRHM	1.515	0.17	5.982	476.01

## 6 Discussion and Conclusions

In this study, a new finite element mesh regrouping strategy-based hybrid light transport model was proposed for XLCT. Based on the luminescence properties of Eu_2_O_3_, according to the optical properties of the mouse model tissues at the emission wavelengths of 610 and 630 nm, adipose, stomach, and kidneys are suitable to adopt the DE approximate modeling, while the heart, liver, and lungs are suitable to adopt the *SP*_3_ approximate modeling. By dividing tissues into two different regions, adipose, stomach, and kidneys belonged to Region1 and the heart, liver, and lungs belonged to Region2. The mouse model was discretized by the finite element method, and the nodes and tetrahedrons were rearranged according to the regions that they belonged to. Furthermore, two meshes were formed according to the division of the two regions. DE and *SP*_3_ approximate modeling were used in the two regions, respectively. The two regions had corresponding correlation system matrixes, the two meshes were combined into a regrouping mesh by coupling these two system matrixes, and a hybrid optical transmission model was established.

Numerical simulations included forward and reverse simulations. In forward simulation corresponding to 610 nm emission wavelength, the results of MRHM were closer to those of MC compared with DE, *SP*_3_, and HDSM, and the results of the DE model were obviously different from those of MC. According to the optical parameters corresponding to 610 nm, the liver is a low-scattering high-absorption organ, which is not suitable for the DE approximate model. At 630 nm emission wavelength, the performance of each model was similar, while MRHM was the best at computational accuracy compared with the other models. The inverse simulations drew similar conclusions to the forward simulations. The MRHM-based method performed better in target location, shape recovery, and image contrast, which had well recovered the position and distribution of the true source. The multispectral reconstruction experiment was adopted to alleviate the ill-posedness of source reconstruction caused by the unique spectrally varying attenuation of biological tissue. Thus, the reconstruction results showed that MRHM can work effectively in multispectral source reconstruction.

*In*-*vivo* experiments were applied to verify the better performance of the proposed MRHM method compared with the DE, *SP*_3_, and HDSM methods. Compared with the simulation experiments, the degeneration on the performance of the *in*-*vivo* experiments resulted from several factors, such as measurement noise of the luminescence distribution on the mouse surface, inadequate prior knowledge of the optical properties of the biological tissues, and errors generated in the process of matching 2D optical data to the coordinate system of the 3D volume data. Even though the performance of all algorithms degraded, MRHM was also the best at computational accuracy among the four models.

In terms of time consumption, the system matrix dimension of *SP*_3_ is twice that of DE, whose calculation was complicated and the time cost was high. HDSM had the same system matrix dimension as *SP*_3_ and complex solution process, leading to still high cost. Furthermore, MRHM was used to reduce the system matrix dimension, which saved 83.4% of the system matrix construction time compared with *SP*_3_ and reduced 86.9% of the inverse calculation time compared with HDSM at 610 nm, and MRHM saved 84.4% of the system matrix construction time compared with *SP*_3_ and reduced 84.9% of the inverse calculation time compared with HDSM at 630 nm. Simultaneously, in reconstruction experiments at 610 and 630 nm, multispectral reconstruction experiment, and *in*-*vivo* experiment, compared with *SP*_3_ and HDSM, MRHM significantly saved over 70% and 95% construction time of the sensitivity matrix, respectively. The advantage of the proposed MRHM method will be more significant with the increased size of computation matrix as the meshes become intensive.

Compared with light transmission models proposed in previous studies, MRHM has several distinguished advantages. Firstly, based on the finite element mesh regrouping strategy, two separate meshes can be obtained. Thus, for the DE and *SP*_3_ models, the system matrixes and source weight matrixes can be calculated separately into two respective mesh systems. Meanwhile, some parallel computation strategy can be combined with finite element mesh regrouping strategy to further save the system matrix calculation time. Secondly, the proposed method can reduce the computational memory consumption than the previously proposed hybrid light transport model achieving good balance between computational accuracy and efficiency. Lastly, the finite element mesh regrouping strategy is a generic framework, which can be used to construct some more accurate hybrid light transport models, such as DE and *SP*_5_, *SP*_3_ and *SP*_5_.

In conclusion, we proposed a new finite element mesh regrouping strategy-based hybrid light transport model for XLCT. Numerical simulations and mouse-based experiments evaluated the accuracy and efficiency of this method. Compared with DE, *SP*_3_, and HDSM, MRHM achieved a balance between computational accuracy and efficiency in optical transmission. It is believed that this novel method will further benefit various preclinical applications of XLCT and facilitate the development of optical molecular tomography in theoretical study.

## Data Availability Statement

The original contributions presented in the study are included in the article/supplementary material. Further inquiries can be directed to the corresponding authors.

## Ethics Statement

The animal study was reviewed and approved by the Animal Ethics Committee of the Northwest University of China.

## Author Contributions

YQL contributed to the design and implementation of this research and successfully achieved the expected goal. XGH was responsible for the data collection, beautification of the illustration, and perfection in the presentation of the manuscript. MXC made some contribution in data collection and in the experiments. HBG provided great help on the whole scheme design of this research and the final article. XWH and JJY provided the research platform with high requirements and rendered some important ideas during our research. All authors contributed to the article and approved the submitted version.

## Funding

This study was funded by the National Natural Science Foundation of China under Grants 61971350, 61901374, 61906154, and 11871321; Natural Science Foundation of Shaanxi under Grant 2019JQ-724; Postdoctoral Innovative Talents Support Program under Grant BX20180254; Scientific and Technological Projects of Xi’an under Grant 201805060ZD11CG44; Key Research and Development Program of Shaanxi 2020SF-036; and Xi’an Science and Technology Project 2019218214GXRC018CG019-GXYD18.3.

## Conflict of Interest

The authors declare that the research was conducted in the absence of any commercial or financial relationships that could be construed as a potential conflict of interest.

## Publisher’s Note

All claims expressed in this article are solely those of the authors and do not necessarily represent those of their affiliated organizations, or those of the publisher, the editors and the reviewers. Any product that may be evaluated in this article, or claim that may be made by its manufacturer, is not guaranteed or endorsed by the publisher.
